# Standard-of-care vs expert-recommended discharge destinations for geriatric surgical inpatients: a prospective observational cohort study

**DOI:** 10.1007/s41999-025-01382-x

**Published:** 2025-12-19

**Authors:** Christoph Leinert, Simone Brefka, Marina L. Fotteler, Annabel S. Mueller-Stierlin, Florian Gebhard, Nuh Rahbari, Christian Bolenz, Hans Kestler, Dhayana Dallmeier, Michael Denkinger, Thomas D. Kocar

**Affiliations:** 1 Institute for Geriatric Research at AGAPLESION Bethesda Ulm and Geriatric Center Ulm, Ulm University Medical Center, Ulm, Germany; 2 Institute for Epidemiology and Medical Biometry, Ulm University, Ulm, Germany; 3 Department for Orthopedic Trauma, Ulm University Medical Center, Ulm, Germany; 4 Department of General and Visceral Surgery, Ulm University Medical Center, Ulm, Germany; 5 Department of Urology, Ulm University Medical Center, Ulm, Germany; 6 Institute of Medical Systems Biology, Ulm University, Ulm, Germany; 7 Department of Epidemiology, Boston University School of Public Health, Boston, MA USA

**Keywords:** Discharge planning, Geriatric co-management, Post-acute care, Functional decline, Readmission

## Abstract

**Purpose:**

Discharge planning is important to ensure optimal postoperative outcomes for older surgical inpatients. As part of the Supporting SURgery with GEriatric co-management and AI (SURGE-Ahead) project, this study investigates how congruence between standard of care discharge decisions and geriatric expert recommendations affects functional outcomes in older surgical inpatients.

**Methods:**

A prospective observational cohort study was conducted across three surgical departments at Ulm University Medical Center (Trauma, Visceral, and Urology). Patients aged 70 years or older with an Identification of Seniors at Risk score ≥ 2 were enrolled. The congruence between the standard of care discharge decisions (actual discharge destination) and recommendations made by expert geriatricians (unknown to clinicians) was determined across four discharge options: home, acute geriatric care unit, post-acute rehabilitation facility, or nursing home. Multivariable logistic regression was employed to examine how the match between recommended and actual discharge destinations related to functional outcomes and readmission rates post-discharge.

**Results:**

Among the 169 participants (mean age 80.5 ± 6.3 years, 58.6% female), a discrepancy of 27% was observed between the standard of care and expert recommendations. Patients with discharge decisions incongruent to geriatric expert recommendations showed higher frailty scores, more dependence in activities of daily living, and reduced mobility pre-operatively. Mismatch between expert recommendations and standard of care was associated with a decline in Barthel Index and Charité Mobility Index scores, and higher 3-month readmission rates.

**Conclusion:**

Optimizing discharge destinations may prevent functional decline and reduce readmissions. Closing the gap between standard of care discharge decisions and geriatric expert recommendations could improve functional outcomes for older surgical inpatients.

**Trial registration:**

German clinical trials registry (DRKS00030684), registered on 21st November 2022.

**Supplementary Information:**

The online version contains supplementary material available at 10.1007/s41999-025-01382-x.

## Key summary points

**Aim:**

Assessment of the congruence between standard discharge decisions and geriatric expert recommendations in older surgical inpatients.

**Findings:**

A 27% discrepancy was observed between standard discharge decisions and geriatrician recommendations, with incongruence associated with higher frailty, greater pre-operative functional limitations, and poorer post-discharge functional outcomes and readmission rates.

**Message:**

Aligning discharge decisions with geriatric expertise may improve functional outcomes and reduce readmissions in older surgical patients.

**Supplementary Information:**

The online version contains supplementary material available at 10.1007/s41999-025-01382-x.

## Introduction

Functional decline of hospitalized older adults is a common concern affecting 20–60% of patients [1–3]. A comprehensive geriatric assessment (CGA) is a multidimensional assessment tool that aims to identify individual treatment and care requirements, initiating tailored interventions and management plans that ultimately aim to prevent functional decline, improving survival, and allowing more patients to live at home 3 to 12 months after discharge [4]. Interdisciplinary care models have been developed to transfer these principles through geriatric co-management into non-geriatric settings such as surgery [5] and have shown to improve outcomes in several settings [6]. Functional decline in geriatric inpatients can be attributed to several factors, including underlying frailty, the trajectory of their acute illness, and organizational aspects of hospital care and discharge [7]. Iatrogenic disability, which refers to avoidable dependence that occurs in the context of hospitalization, acts as a modifiable risk factor contributing to the functional decline commonly observed in older patients [7]. Effective discharge planning, an important aspect of geriatric co-management, has been shown to reduce readmission rates [8] and to increase discharge to favorable settings like post-acute rehabilitation facilities [9] or home [10].

Due to demographic trends, the number of older people treated in surgical departments is rising, leading to a growing need for geriatric co-management. However, limitations in the availability of qualified geriatricians pose ongoing challenges to meeting this requirement [11]. Recognizing these limitations, the Supporting SURgery with GEriatric co-management and artificial intelligence (SURGE-Ahead) project was initiated to develop an AI-supported clinical decision support system (CDSS), assisting with inpatient geriatric co-management and providing recommendations on optimal postoperative care pathways [12].

Within the SURGE-Ahead project, we conducted the observational and AI development study with a prospective observational cohort design in three surgical departments at Ulm University Medical Center (trauma surgery, general and visceral surgery, urology). This study aimed to collect data on the geriatric patient population to facilitate the development of AI algorithms and provide a comparative framework for future interventions. Additionally, it evaluated the need for geriatric co-management and compared the existing standard of care regarding discharge destinations with the recommendations of independent geriatric experts [12]. The present article outlines the methodology and findings of the SURGE-Ahead observational and AI development study.

## Methods

### Data collection

**Fig. 1:**
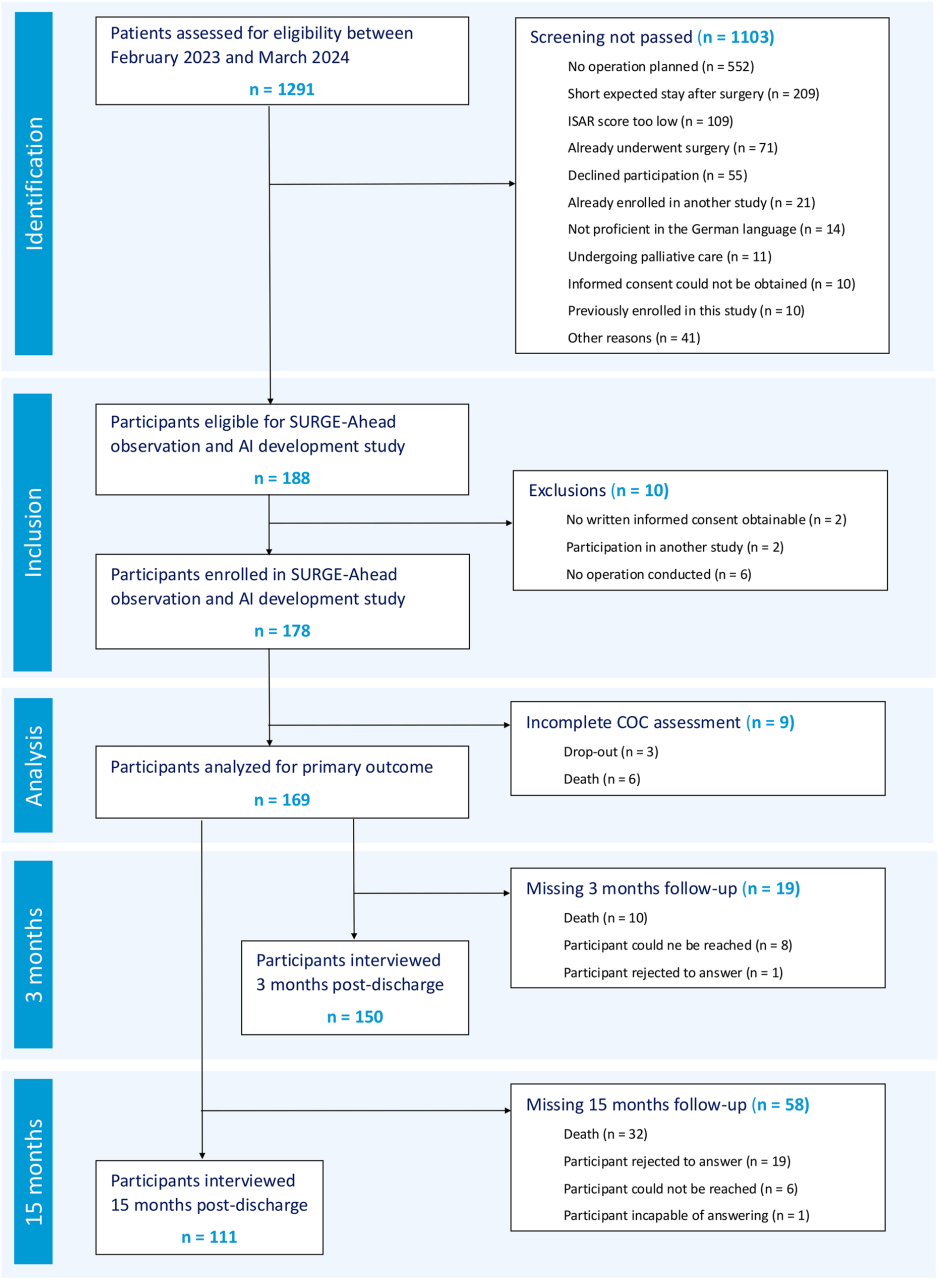
STROBE flow chart. Out of 1291 patients that were assessed for eligibility in the SURGE-Ahead observational and AI development study, 178 were successfully enrolled. Nine patients did not reach the primary outcome, either due to dropout (*n* = 3) or death (*n* = 6), leading to a final analysis cohort of 169 patients. In the 3- and 15-month follow-up, 19 and 58 participants could not be interviewed, respectively. *ISAR* identifying seniors at risk

The SURGE-Ahead observational and AI development study took place from February 2023 to March 2024 in the trauma surgery, general and visceral surgery, and urology departments of Ulm University Medical Center. Detailed information can be found in the study protocol [12]. Our study included patients aged 70 years or older who were admitted for surgical intervention at one of the three participating departments, had not undergone surgery yet, and had an Identification of Seniors at Risk (ISAR) score of 2 or higher [13]. Exclusion criteria comprised patients with a life expectancy of less than 3 months, those unable to provide informed consent and without a legal guardian, individuals with limited communication abilities, participants in other interventional studies, and patients expected to stay less than three nights. To avoid selection bias, weekend and evening shifts were implemented for recruitment, ensuring full coverage of all patients admitted to Ulm University Medical Center. Out of 1291 patients screened for the study, 1103 were deemed ineligible primarily due to no planned surgical intervention (*n* = 552), anticipated hospital stays of less than three nights (*n* = 209), and ISAR scores lower than 2 (*n* = 109). Of the 178 participants enrolled, 9 did not reach the primary outcome of being discharged from their respective departments, due to drop-out (*n* = 3) and death (*n* = 6). In the 3- and 15-month follow-up, 19 and 58 participants could not be interviewed, respectively. For a STROBE chart, see Fig. 1.

### Explanatory variables

We considered several key explanatory variables related to the patient’s functional characteristics in the multiple dimensions of a minimum geriatric data set (MGDS) [12] and the surgical procedure. These included age (years), sex (male/female), and whether the patient underwent trauma surgery (yes/no), general and visceral surgery (yes/no), or urology (yes/no). Additionally, we examined body mass index (BMI; kg/m^2^), Nutritional Risk Screening (NRS; positive/negative [14]), Montreal Cognitive Assessment 5-min protocol (MoCA 5-min; score [15, 16]), presence of dementia (yes/no), Patient Health Questionnaire-4 (PHQ-4; score [17]), ISAR Screening (score [13]), Clinical Frailty Scale (CFS; score [18]), American Society of Anesthesiologists score (ASA; class I–V [19]), the total number of medications taken (n), care level (class I–V), nursing services received (none, at home, assisted living, nursing home), living alone status (yes/no), Barthel Index (activities of daily living (ADL) score [20]), Charité Mobility Index (CHARMI; score) [21], New Mobility Score (NMS; score [22]), history of falls within the last 3 months (yes/no), emergency surgery status (yes/no), time from admission to surgery (minutes), cut-to-suture time (minutes), length of stay in the intensive care unit (minutes), and overall length of hospital stay (days).

German MoCA subtest were derived from the validated German Version 8.1 of the full MoCA test provided by www.mocacognition.org, with the exception of the verbal fluency subtest that has been translated separately [16]. For the purpose of this analysis, the Barthel Index was used as the sole measure of ADL.

### Outcomes

In this observational study, the primary outcome measured was the congruence of the discharge destination between standard of care decisions (actual discharge decision made by the treating surgical team) and geriatric expert recommendations. Discharge destinations were categorized into four classes: home, acute geriatric care unit, post-acute rehabilitation facility, and nursing home. (A description of the acute and post-acute geriatric care facilities available in the regional study setting of southwest Germany is provided by Becker and colleagues [23]). Congruence was determined by comparing the standard of care decision to the judgment of two experienced geriatricians. Each patient received at least one visit from an expert geriatrician during their inpatient stay, occurring within 3 days before discharge. During these visits, geriatricians documented their recommended discharge destination, which was not visible to the treating clinicians. This recommendation considered: impressions during personal visit as well as MGDS and routine care data to assess post-acute rehabilitation eligibility, medical and nursing care needs as well as the personal preferences of the patient. At the end of the study, both geriatricians reviewed each case and reassessed the suggested discharge destination based on the additional 3-month follow-up data and a thorough review of the patient's medical chart.

Secondary outcomes were evaluated through follow-up interviews conducted 3 and 15 months post-discharge. These included assessments of care level, institutionalization status (change in accommodation to a nursing home), Barthel Index score (for ADL), CHARMI score, and New Mobility Score (for mobility). Additionally, during the 3-month follow-up interview, data on nursing services received (none, at-home care, assisted living, or nursing home) and any hospital readmissions (yes/no) were recorded, the latter supplemented by data from the electronic patient record.

### Statistical analysis

Secondary outcomes were dichotomized based on their change from preoperative to follow-up status, as follows: improvement/no change (coded as 0) versus deterioration (coded as 1). Descriptive statistics were calculated for each explanatory variable and secondary outcome, stratified by the primary outcome, i.e., the congruence of the discharge destination. Means and standard deviations (SD) are reported for continuous variables, while frequencies and percentages are presented for binary variables. Additionally, exploratory analyses were performed to investigate potential group differences. For continuous or discrete explanatory variables, either Student’s *t* tests or Mann–Whitney U tests were employed, depending on the normality of the data. The Mann–Whitney U test was used for ordinal explanatory variables, while chi-square tests with Yates’ continuity correction were applied for binary explanatory variables. Secondary outcomes were modeled using logistic regression analyses, with the congruence of the discharge destination included as a predictor variable, while adjusting for relevant covariates identified by significant group differences in the exploratory analyses. All statistical analyses have been conducted using the scipy 1.12 library and the statsmodel 0.14 library for Python [24, 25]. p-values smaller than 0.05 were considered statistically significant. No correction for multiple comparisons was conducted, as this was an exploratory analysis.

### Ethics and consent statement

The observational and AI development study of the SURGE-Ahead project was conducted following ethical guidelines set by the Declaration of Helsinki. It was approved by the University of Ulm’s Ethical Committee with the reference number # 310/22-Sta. All participants provided their written informed consent.

## Results

### Study population

The study included 169 older patients, consisting of 70 (41.4%) males and 99 (58.6%) females, with a mean age of 80.5 years (SD ± 6.3). Most patients underwent trauma surgery (75.1%), while general and visceral surgery, and urology accounted for a smaller percentage of cases. The mean BMI was 26.3 kg/m^2^ (SD ± 5.0). Cognitive impairment was assessed using the MoCA 5-min score, averaging 21.7 (SD ± 5.9), with 11.2% of participants previously diagnosed with dementia. The PHQ-4 score indicated moderate anxiety and depression levels, averaging 2.5 (SD ± 2.7). The mean ISAR score was 3.0 (SD ± 1.0), suggesting a moderate risk of adverse functional outcomes [13]. Patients received an average of 8.8 medications (SD ± 3.8), and nearly half lived alone preoperatively. Functional capacity was evaluated using the Barthel Index, scoring 86.1 (SD ± 19.2) on average. A majority experienced previous falls, and more than half underwent emergency surgery. The mean time to surgery was 62.6 h (SD ± 87.6) across specialties: trauma surgery (67.7 ± 92.0), general and visceral surgery (69.5 ± 94.2), urology (22.8 ± 8.9). The average cut-to-suture time was 89.7 min (SD ± 67.3). The average length of ICU and hospital stay per patient were 409 min (SD ± 977) and 12.4 days (SD ± 10.3), respectively. For more details, see Table 1.

**Table 1:** Study population characteristics

	COC congruent (*n* = 124)		COC incongruent (*n* = 45)		Group difference
	Mean (± SD)	*n* (%)	Mean (± SD)	*n* (%)	*p* value
Age (years)	79.8 (± 6.2)	–	82.3 (± 6.2)	–	**0.0075**
Sex (male)	–	52 (41.9%)	–	18 (40.0%)	0.9608
Sex (female)	–	72 (58.1%)	–	27 (60.0%)	–
Trauma surgery (yes/no)	–	89 (71.8%)	–	38 (84.4%)	0.1380
General and visceral surgery (yes/no)	–	20 (16.1%)	–	2 (4.4%)	0.0824
Urology (yes/no)	–	15 (12.1%)	–	5 (11.1%)	1.0000
BMI (kg/m^2^)	26.4 (± 4.6)	–	25.8 (± 6.2)	–	0.1913
NRS (yes/no)	–	51 (41.1%)	–	28 (62.2%)	**0.0112**
MoCA 5-min (score)	21.4 (± 6.1)	–	22.6 (± 5.2)	–	0.4129
Dementia (yes/no)	–	13 (10.5%)	–	6 (13.3%)	0.8081
PHQ4 (score)	2.5 (± 2.7)	–	2.3 (± 2.8)	–	0.4125
ISAR (score)	2.9 (± 0.9)	–	3.4 (± 0.9)	–	**0.0008**
CFS (score)	4.0 (± 1.8)	–	4.9 (± 1.7)	–	**0.0018**
ASA (class)	2.9 (± 0.5)	–	3.1 (± 0.5)	–	**0.0165**
Number of medications (n)	8.5 (± 3.8)	–	9.8 (± 3.7)	–	**0.0430**
Care level (class)	0.8 (± 1.2)	–	1.4 (± 1.4)	–	**0.0150**
Nursing services (class)	1.1 (± 0.7)	–	1.2 (± 0.8)	–	0.2286
Living alone (yes/no)	–	56 (45.2%)	–	22 (48.9%)	0.7986
Barthel Index (score)	87.6 (± 19.0)	–	82.1 (± 19.2)	–	**0.0260**
CHARMI (score)	8.1 (± 2.5)	–	7.4 (± 3.1)	–	0.2412
NMS (score)	6.6 (± 2.5)	–	5.5 (± 2.3)	–	**0.0071**
Falls (yes/no)	–	71 (57.3%)	–	33 (73.3%)	0.1041
Emergency surgery (yes/no)	–	67 (54%)	–	28 (62.2%)	0.4394
Time to surgery (h)	60.6 (± 93.4)	–	68.1 (± 69.5)	–	0.2054
Cut-to-suture time (min)	92.3 (± 69.7)	–	82.5 (± 60.0)	–	0.4201
Length of stay ICU (min)	366 (± 619)	–	531 (± 1603)	–	0.3233
Length of hospital stay (days)	10.8 (± 7.7)	–	17.0 (± 14.5)	–	**0.0006**

Significant differences are highlighted in bold

*BMI* body mass index, *NRS* nutritional risk screening, *MoCA* Montreal Cognitive Assessment, *PHQ4* Patient Health Questionnaire-4, *ISAR* identifying seniors at risk screening, *CFS* Clinical frailty scale, *ASA* American Society of Anesthesiologists score, *CHARMI* Charité mobility index, *NMS* New Mobility Score, *ICU* intensive care unit

### Primary outcome

**Fig. 2:**
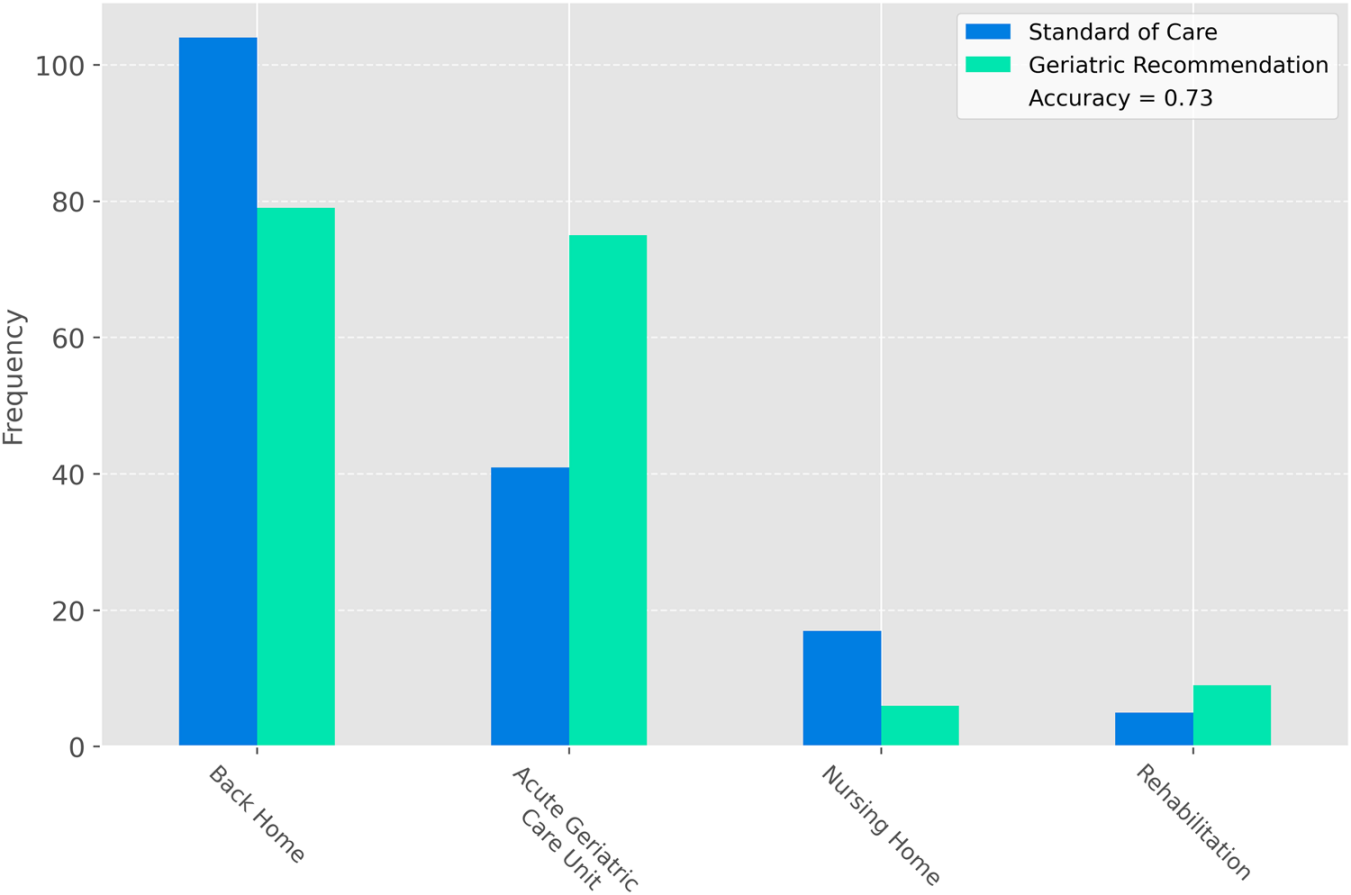
Primary outcome. More patients were discharged home and fewer to acute geriatric care units, differing from geriatric expert recommendations. Standard of care accuracy: 0.73. *Rehabilitation*  post-acute rehabilitation facility

The standard of care discharge destination was congruent with the geriatric expert recommendations in 73% of the cases (see Fig. 2). Overall, more patients were discharged back home and less to an acute geriatric care unit compared to the expert recommendation. Different explanatory variables were associated with the congruence between the standard of care and the expert-recommended discharge destination (see Table 1). Significant differences were found for age, NRS, ISAR screening, CFS, ASA score, number of medications, care level, Barthel Index, NMS, and length of hospital stay, serving as covariates for subsequent analyses.

### Secondary outcomes

A mismatch between the expert recommendation and the actual discharge decision was associated with worse functional outcomes for the Barthel Index, CHARMI, and readmissions at 3 months while controlling for all relevant covariates (see Table 2). Most outcomes revealed trends in the same directions at 3 and 15 months, but none of the others reached statistical significance.

**Table 2:** Secondary outcomes

	COC congruent (*n* = 124)	COC incongruent (*n* = 45)	Missing (*n*, %)	Odds ratio [95% CI]	Group difference (*p* values)
3-month follow-up					
Care level increase (3 months)	14 (11.3%)	10 (22.2%)	21 (12.4%)	0.47 [0.13–1.72]	0.2563
Nursing services increase (3 months)	14 (11.3%)	2 (4.4%)	21 (12.4%)	1.54 [0.24–9.93]	0.6519
Barthel index decline (3 months)	29 (23.4%)	22 (48.9%)	23 (13.6%)	**0.35 [0.12–0.99]**	**0.0494**
CHARMI decline (3 months)	26 (21%)	16 (35.6%)	37 (21.9%)	**0.31 [0.10–0.91]**	**0.0326**
NMS decline (3 months)	41 (33.1%)	22 (48.9%)	22 (13.0%)	0.62 [0.22–1.80]	0.3829
Readmission (3 months)	12 (9.7%)	11 (24.4%)	0 (0.0%)	**0.13 [0.04–0.50]**	**0.0027**
Institutionalization (3 months)	3 (2.4%)	0 (0.0%)	20 (11.8%)	NA [0.00–inf]	0.9990
15-month follow-up					
Care level increase (15 months)	19 (15.3%)	10 (22.2%)	63 (37.3%)	0.40 [0.11–1.42]	0.1549
Barthel index decline (15 months)	34 (27.4%)	15 (33.3%)	59 (34.9%)	0.48 [0.12–1.88]	0.2904
CHARMI decline (15 months)	15 (12.1%)	6 (13.3%)	73 (43.2%)	1.29 [0.27–6.01]	0.7499
NMS decline (15 months)	27 (21.8%)	13 (28.9%)	59 (34.9%)	0.52 [0.15–1.83]	0.3045
Institutionalization (15 months)	2 (1.6%)	0 (0.0%)	58 (34.3%)	0.52 [0.15–1.83]	NA

Group differences, including odds ratios, were corrected for explanatory variables that were significantly different between groups are highlighted in bold

*COC* continuity of care, *CHARMI* Charité Mobility Index, *NMS* New Mobility Score, *Institutionalization* change in accommodation to a nursing home, *NA* no result due to small sample sizes

## Discussion

The present study revealed a mismatch of 27% between standard of care and expert geriatricians’ recommended discharge destination. The experts recommended the transfer to an acute geriatric care unit or post-acute rehabilitation facility more frequently, and less frequently to a nursing home or directly home. Incongruence in discharge decisions was associated with increased frailty, more dependence in ADL, lower mobility before admission, and a longer length of stay in the hospital. At the 3-month follow-up, functional decline in ADL and mobility as well as a higher readmission rate were associated with incongruent discharge decisions.

Hospitalization of older adults is considered an important turning point in their health trajectory [26]. As we only included patients with an ISAR score of 2 or higher, our cohort contained a high proportion of frail older patients coming close to a real-world geriatric population with a high risk of functional decline as well as a higher rate of discharge to nursing homes [27]. The treatment of older patients in a specialized geriatric setting can prevent functional decline [28], promote functional recovery, and reduce mortality [29], whereas the choice of a non-specialized setting after an acute event might contribute to iatrogenic disability [30, 31]. The effects on mobility and ADL observed at 3 months were not sustained through the 15-month follow-up, likely due to the limited sample size and potential for recovery over time. Nevertheless, other studies have shown that the failure to regain function after 3 months predicts even further deterioration with a higher rate of institutionalization 12 months after discharge [32].

Our findings demonstrate that alignment with geriatric expert recommendations during discharge planning is associated with improved functional outcomes and reduced readmission rates, reinforcing the established benefits of comprehensive discharge planning [8]. While our results are situated within the German healthcare system, a recent scoping review [33] identified consistent predictor domains—cognitive function, functional status, and social support—across diverse healthcare settings. This suggests that the principle of optimizing discharge destinations based on individualized patient needs is broadly applicable, even with variations in resource availability and healthcare infrastructure. Successful interventions for the prevention and treatment of functional decline in acutely hospitalized frail older adults include functional exercise programs [34–36]. Many health care systems provide post-acute rehabilitation programs, offering this kind of exercise and the possibility of functional recovery for older adults, e.g., acute care geriatric units or post-acute rehabilitation services in Germany [23], Italy [31], or Australia [37]. As discharge options always depend on the respective health care system, the generalizability of our results is limited. Nevertheless, the choice of the optimal discharge destination is challenging in all different health care settings, and comparable options to our investigation can be found in many other health care systems.

Besides discharge options offering functional recovery, many healthcare systems struggle with ‘capacity pressure’ in admissions, a phenomenon where systemic constraints reduce hospital intake capacity, leading to delayed admissions and impacting patient care timelines [38]. ‘Bed blockage’, primarily concerning post-treatment patient flow issues, directly contributes to this pressure by delaying patient discharge and reducing bed turnover, thereby worsening admission wait times [38]. In the present study, the mean length of stay in the group with congruent discharge decisions was much shorter compared to the group with incongruent discharge decisions (10.8 vs 17 days). In the context of our study, this indicates that ‘capacity pressure’ was probably not the main driver of the incongruence. Another possible reason for incongruence could have been limited access to acute geriatric or post-acute care in our study. Even though the study region is well-equipped with acute- and post-acute geriatric beds, temporary constraints may have hindered transfers. As data on discharge barriers were not collected, further research is needed to determine the reasons for the differences between expert recommendations and actual discharge destinations and observed in this study.

Another important part of geriatric treatment and discharge planning, considering the concept of ‘shared decision-making’, is the assessment of patient preferences, as there are clear individual differences here [39]. Respecting patient autonomy in the context of discharge planning can be challenging and can lead to an ethical dilemma when professional recommendations differ from patient wishes [40]. The value of a patient’s preference and personal goals was also taken into account by the expert geriatricians in our study when determining their recommendation for the optimal discharge destination. We emphasize the importance of considering patient preferences and personal goals in standard of care discharge planning.

Geriatric co-management typically involves a multicomponent intervention grounded in CGA and multidisciplinary team collaboration. The inherent complexity of this intervention often obscures the relative contribution of individual components, effectively creating a “black box” effect [41]. Our study demonstrates that the single component of the individual decision regarding post-discharge placement could impact meaningful patient outcomes—such as impairments in ADL and mobility. This underscores the critical role of geriatric expertise not only during acute surgical care but also in discharge planning. The discrepancy of the standard of care and expert recommendations opens up potential for improvement through a CDSS. In a recent study within the SURGE-Ahead project, health care professionals were interviewed concerning their expectations and requirements for a CDSS for the co-management of older adults [42]. The decision concerning the optimal post-surgical care option was described as one major challenge in the treatment of geriatric patients. Other studies describe that evaluation processes for post-acute rehabilitation eligibility are poorly understood and lack objective parameters [43].

The evaluation of the AI-based CDSS within the SURGE-Ahead project demonstrates potential benefits for geriatric perioperative care, but also highlights critical ethical and practical considerations, including data privacy, algorithmic bias, and ensuring transparency. Future models must prioritize incorporating patient values and preferences [44]. Emerging technologies, such as sensor-based data acquisition utilizing inertial measurement units (IMUs), offer promising avenues for automated assessment and personalized discharge planning. Notably, in a recent publication we demonstrated that IMU data outperforms standard of care accuracy in suggesting suitable discharge destinations [45]. Stakeholder perspectives consistently emphasized the need to balance AI’s potential to improve holistic assessment and decision-making with concerns regarding the patient-physician relationship, data security, and algorithmic bias [42, 44, 46, 47]. These findings underscore the importance of responsible AI implementation, prioritizing usability, human oversight, and transparent governance to ensure equitable and beneficial integration into geriatric healthcare.

While prior research demonstrates the benefits of geriatric co-management in discharge planning [8], our study uniquely identifies specific discharge destinations—acute geriatric care, post-acute rehabilitation, nursing home, and direct home discharge—as key therapeutic targets. As a next step in the SURGE-Ahead project, this insight informs our ongoing intervention trial, leveraging a machine learning algorithm to address the incongruence between standard surgical discharge decisions and geriatric recommendations by providing a CDSS suggestion for optimal discharge destinations, ultimately aiming to improve patient outcomes.

We identified several limitations for this prospective observational cohort study. First, as this was an exploratory analysis, no adjustments for multiple testing were performed. It is important to acknowledge that significant differences observed may be due to chance because of multiple comparisons. Second, the small sample size (*n* = 169) constrains the generalizability of our findings. The wide confidence intervals observed for the estimated effect sizes highlight the potential for variability across different populations. Third, a potential limitation of this study was the high rate of missing data observed during the 15-month follow-up assessment, which may have contributed to our inability to detect statistically significant differences, as shown in Table 2. Fourth, the standard of care discharge decision might have been modified by the availability of certain discharge options or due to organizational restrictions like bed capacities, or the delay of cost coverage confirmations for post-acute rehabilitation services. These issues might have led in part to discharge decisions that were incongruent with the geriatric expert’s recommendation. Due to the observational design of the study, we were unable to directly interview stakeholders involved in discharge decision-making to ascertain their rationale or identify potential barriers. We plan to carefully evaluate these potential barriers through direct stakeholder engagement during an intervention trial currently conducted at the study centers. Fifth, this study is limited by its observational design and the German healthcare context, potentially affecting generalizability. The observed length of stay exceeds that reported in surgical departments across many European nations. This discrepancy is likely attributable to the German Diagnosis-Related Group (DRG) system, which incentivizes thoroughness and comprehensive comorbidity management rather than expedited discharge [48]. However, a recent scoping review [33] suggests that common predictor domains for discharge destination may mitigate concerns regarding limited generalizability. Sixth, while ‘living alone’ served as a pragmatic proxy for social frailty in this study, it is recognized that a more comprehensive assessment of social vulnerability is preferred when addressing this complex construct in an academic context. Seventh, our study data did not explicitly encompass patient preferences concerning discharge destinations. Nevertheless, we implicitly included patient preferences in the expert recommendations. In this study, patient preferences are systematically recorded and displayed to surgeons to facilitate alignment with patient values. Eighth, adjusting for covariates may introduce the risk of multicollinearity between the explanatory variable and the confounders. Fortunately, this was not the case in our study (see supplementary Table 1**)**. Finally, to investigate whether insufficient healthcare provision was the main driver of poorer patient outcomes in the 3- and 15-month follow-up, we repeated the analysis without patients that were overtreated, according to the expert discharge destination, i.e., discharged to a place where they received more care than they actually needed (*n* = 2). For the secondary outcomes readmission (3 months), institutionalization (3 months), care level (15 months), and CHARMI (15 months), *p* values were the same or decreased. In all other secondary outcomes, *p* values increased, suggesting insufficient healthcare provision being a confounding factor in these instances. However, in clinical practice, this distinction may be of little relevance, as geriatric co-management is mostly needed in cases where insufficient healthcare provision may not be identified.

## Conclusion

The present study aimed to assess the congruence of standard of care discharge decisions and geriatric expert recommendations for geriatric surgical inpatients. Results showed a gap of 27% between standard of care and expert recommendations, with incongruent discharge decisions leading to a higher rate of readmissions as well as increased dependence in ADL and reduced mobility in a 3-month follow-up. These findings highlight the importance of optimizing discharge planning for geriatric surgical inpatients to prevent functional decline and will guide the development of a CDSS within the SURGE-Ahead project.

## Supplementary Information

Below is the link to the electronic supplementary material.

Supplementary file1 (XLSX 29 KB)

## Data Availability

Data used in this specific analysis can also be accessed on the project’s GitHub page: https://github.com/IfGF-UUlm/SA_OKIE

## References

[1] Arnau A, Espaulella J, Serrarols M, Canudas J, Formiga F, Ferrer M (2016) Risk factors for functional decline in a population aged 75 years and older without total dependence: a one-year follow-up. Arch Gerontol Geriatr 65:239–247. 10.1016/j.archger.2016.04.002 27131227 10.1016/j.archger.2016.04.002

[2] Boyd CM, Landefeld CS, Counsell SR, Palmer RM, Fortinsky RH, Kresevic D et al (2008) Recovery of activities of daily living in older adults after hospitalization for acute medical illness. J Am Geriatr Soc 56:2171–2179. 10.1111/j.1532-5415.2008.02023.x 19093915 10.1111/j.1532-5415.2008.02023.x PMC2717728

[3] Chang H-H, Tsai S-L, Chen C-Y, Liu W-J (2010) Outcomes of hospitalized elderly patients with geriatric syndrome: report of a community hospital reform plan in Taiwan. Arch Gerontol Geriatr 50(Suppl 1):S30-33. 10.1016/S0167-4943(10)70009-1 20171453 10.1016/S0167-4943(10)70009-1

[4] Ellis G, Gardner M, Tsiachristas A, Langhorne P, Burke O, Harwood RH et al (2017) Comprehensive geriatric assessment for older adults admitted to hospital. Cochrane Database Syst Rev 9:CD006211. 10.1002/14651858.CD006211.pub3 10.1002/14651858.CD006211.pub3 PMC6484374 28898390

[5] Rapp K, Becker C, Todd C, Rothenbacher D, Schulz C, König H-H et al (2020) The association between orthogeriatric co-management and mortality following hip fracture. Dtsch Arztebl Int 117:53–59. 10.3238/arztebl.2020.0053 32036854 10.3238/arztebl.2020.0053 PMC7036469

[6] Fierbinţeanu-Braticevici C, Raspe M, Preda AL, Livčāne E, Lazebnik L, Kiňová S et al (2019) Medical and surgical co-management – a strategy of improving the quality and outcomes of perioperative care. Eur J Intern Med 61:44–47. 10.1016/j.ejim.2018.10.017 30448097 10.1016/j.ejim.2018.10.017

[7] Lafont C, Gérard S, Voisin T, Pahor M, Vellas B (2011) The members of I.A.G.G. / A.M.P.A Task Force. Reducing “iatrogenic disability” in the hospitalized frail elderly. J Nutr Health Aging 15:645–660. 10.1007/s12603-011-0335-7 21968859 10.1007/s12603-011-0335-7 PMC12878894

[8] Gonçalves-Bradley DC, Lannin NA, Clemson L, Cameron ID, Shepperd S (2022) Discharge planning from hospital. Cochrane Database Syst Rev 2:CD000313. 10.1002/14651858.CD000313.pub6 10.1002/14651858.CD000313.pub6 PMC8867723 35199849

[9] Raijmann RCMA, Koek HL, Emmelot-Vonk MH, Swaving JGE, Agema WRP, Kerckhoffs APM et al (2024) Impact of geriatric co-management on outcomes in hospitalised cardiology patients aged 85 and over. Neth Heart J 32:76–83. 10.1007/s12471-023-01806-y 37651030 10.1007/s12471-023-01806-y PMC10834903

[10] Saha S, DiRusso SM, Welle S, Lieberman B, Sender J, Shabsigh R et al (2019) Integration of geriatrician consultation for trauma admissions may benefit patient outcomes. Gerontol and Geriatr Med 5:2333721419858735. 10.1177/2333721419858735 31259206 10.1177/2333721419858735 PMC6589989

[11] Lester PE, Dharmarajan TS, Weinstein E (2020) The looming geriatrician shortage: ramifications and solutions. J Aging Health 32:1052–1062. 10.1177/0898264319879325 31583940 10.1177/0898264319879325

[12] Leinert C, Fotteler M, Kocar TD, Dallmeier D, Kestler HA, Wolf D et al (2023) Supporting SURgery with GEriatric Co-Management and AI (SURGE-Ahead): a study protocol for the development of a digital geriatrician. PLoS ONE 18:e0287230. 10.1371/journal.pone.0287230 37327245 10.1371/journal.pone.0287230 PMC10275448

[13] McCusker J, Bellavance F, Cardin S, Trépanier S, Verdon J, Ardman O (1999) Detection of older people at increased risk of adverse health outcomes after an emergency visit: the ISAR screening tool. J Am Geriatr Soc 47:1229–1237. 10.1111/j.1532-5415.1999.tb05204.x 10522957 10.1111/j.1532-5415.1999.tb05204.x

[14] Schütz T, Valentini L, Plauth M (2005) Screening auf Mangelernährung nach den ESPEN-Leitlinien 2002. Aktuelle Ernahrungsmed 30:99–103. 10.1055/s-2004-834733

[15] Wong A, Nyenhuis D, Black SE, Law LSN, Lo ESK, Kwan PWL et al (2015) Montreal Cognitive Assessment 5-minute protocol is a brief, valid, reliable, and feasible cognitive screen for telephone administration. Stroke 46:1059–1064. 10.1161/STROKEAHA.114.007253 25700290 10.1161/STROKEAHA.114.007253 PMC4373962

[16] Costa AS, Fimm B, Friesen P, Soundjock H, Rottschy C, Gross T et al (2012) Alternate-form reliability of the Montreal cognitive assessment screening test in a clinical setting. Dement Geriatr Cogn Disord 33:379–384. 10.1159/000340006 22797211 10.1159/000340006

[17] Kroenke K, Spitzer RL, Williams JBW, Löwe B (2009) An ultra-brief screening scale for anxiety and depression: the PHQ-4. Psychosomatics 50:613–621. 10.1176/appi.psy.50.6.613 19996233 10.1176/appi.psy.50.6.613

[18] Rockwood K, Song X, MacKnight C, Bergman H, Hogan DB, McDowell I et al (2005) A global clinical measure of fitness and frailty in elderly people. CMAJ 173:489–495. 10.1503/cmaj.050051 16129869 10.1503/cmaj.050051 PMC1188185

[19] Daabiss M (2011) American Society of Anaesthesiologists physical status classification. Indian J Anaesth 55:111–115. 10.4103/0019-5049.79879 21712864 10.4103/0019-5049.79879 PMC3106380

[20] Heuschmann PU, Kolominsky-Rabas PL, Nolte CH, Hünermund G, Ruf H-U, Laumeier I et al (2005) The reliability of the German version of the barthel-index and the development of a postal and telephone version for the application on stroke patients. Fortschr Neurol Psychiatr 73:74–82. 10.1055/s-2004-830172 15685491 10.1055/s-2004-830172

[21] Liebl ME, Elmer N, Schroeder I, Schwedtke C, Baack A, Reisshauer A (2016) Introduction of the Charité Mobility Index (CHARMI) – a novel clinical mobility assessment for acute care rehabilitation. PLoS ONE 11:e0169010. 10.1371/journal.pone.0169010 28006023 10.1371/journal.pone.0169010 PMC5179242

[22] Peter V (2020) Mobilitätsstatus erheben und Prognose abgeben – New Mobility Score. Ergopraxis 13:38–39. 10.1055/a-1101-5177

[23] Becker C, Rapp K, Rothenbacher D, Schulz C, König H-H, Büchele G (2020) Acute care models for hip fracture treatment vs post-acute rehabilitation services in older adults after hip fracture: a comparative claims data analysis from Germany. J Rehabil Med 52:jrm00024. 10.2340/16501977-2630 31748818 10.2340/16501977-2630

[24] Virtanen P, Gommers R, Oliphant TE, Haberland M, Reddy T, Cournapeau D et al (2020) SciPy 1.0: fundamental algorithms for scientific computing in Python. Nat Methods 17:261–272. 10.1038/s41592-019-0686-2 32015543 10.1038/s41592-019-0686-2 PMC7056644

[25] Seabold S, Perktold J (2010) Statsmodels: econometric and statistical modeling with Python. Scipy 7(1):92–96. 10.25080/Majora-92bf1922-011

[26] Martínez-Velilla N, Herrero AC, Cadore EL, de Sáez Asteasu ML, Izquierdo M (2016) Iatrogenic nosocomial disability diagnosis and prevention. J Am Med Dir Assoc 17:762–764. 10.1016/j.jamda.2016.05.019 27349623 10.1016/j.jamda.2016.05.019

[27] Evans SJ, Sayers M, Mitnitski A, Rockwood K (2014) The risk of adverse outcomes in hospitalized older patients in relation to a frailty index based on a comprehensive geriatric assessment. Age Ageing 43:127–132. 10.1093/ageing/aft156 24171946 10.1093/ageing/aft156

[28] Zelada MA, Salinas R, Baztán JJ (2009) Reduction of functional deterioration during hospitalization in an acute geriatric unit. Arch Gerontol Geriatr 48:35–39. 10.1016/j.archger.2007.09.008 18022709 10.1016/j.archger.2007.09.008

[29] Baztán JJ, Gálvez CP, Socorro A (2009) Recovery of functional impairment after acute illness and mortality: one-year follow-up study. Gerontology 55:269–274. 10.1159/000193068 19141990 10.1159/000193068

[30] Sourdet S, Lafont C, Rolland Y, Nourhashemi F, Andrieu S, Vellas B (2015) Preventable iatrogenic disability in elderly patients during hospitalization. J Am Med Dir Assoc 16:674–681. 10.1016/j.jamda.2015.03.011 25922117 10.1016/j.jamda.2015.03.011

[31] Galizia G, Balestrieri G, De Maria B, Lastoria C, Monelli M, Salvaderi S et al (2018) Role of rehabilitation in the elderly after an acute event: insights from a real-life prospective study in the subacute care setting. Eur J Phys Rehabil Med 54:934–938. 10.23736/S1973-9087.18.05221-8 29898588 10.23736/S1973-9087.18.05221-8

[32] Portegijs E, Buurman BM, Essink-Bot M-L, Zwinderman AH, de Rooij SE (2012) Failure to regain function at 3 months after acute hospital admission predicts institutionalization within 12 months in older patients. J Am Med Dir Assoc 13:569.e1-569.e7. 10.1016/j.jamda.2012.04.003 22572555 10.1016/j.jamda.2012.04.003

[33] Leinert C, Fotteler ML, Kocar TD, Wolf J, Beissel L, Grummich K, Dallmeier D, Denkinger M (2025) Identifying key predictors of appropriate discharge destinations for older inpatients in acute care: a scoping review. Interact J Med Res 14:e76582. 10.2196/76582 10.2196/76582 PMC12826578 41570253

[34] Ortiz-Alonso J, Bustamante-Ara N, Valenzuela PL, Vidán-Astiz M, Rodríguez-Romo G, Mayordomo-Cava J et al (2020) Effect of a simple exercise program on hospitalization-associated disability in older patients: a randomized controlled trial. J Am Med Dir Assoc 21:531-537.e1. 10.1016/j.jamda.2019.11.027 31974063 10.1016/j.jamda.2019.11.027

[35] Hartley P, Keating JL, Jeffs KJ, Raymond MJ, Smith TO (2022) Exercise for acutely hospitalised older medical patients. Cochrane Database Syst Rev 11:CD005955. 10.1002/14651858.CD005955.pub3 36355032 10.1002/14651858.CD005955.pub3 PMC9648425

[36] Martínez-Velilla N, Casas-Herrero A, Zambom-Ferraresi F, de Sáez Asteasu ML, Lucia A, Galbete A et al (2019) Effect of exercise intervention on functional decline in very elderly patients during acute hospitalization: a randomized clinical trial. JAMA Intern Med 179:28–36. 10.1001/jamainternmed.2018.4869 30419096 10.1001/jamainternmed.2018.4869 PMC6583412

[37] Mitchell R, Harvey L, Draper B, Brodaty H, Close J (2017) Risk factors associated with residential aged care, respite and transitional aged care admission for older people following an injury-related hospitalisation. Arch Gerontol Geriatr 72:59–66. 10.1016/j.archger.2017.05.012 28599139 10.1016/j.archger.2017.05.012

[38] Ekdahl AW, Linderholm M, Hellström I, Andersson L, Friedrichsen M (2012) Are decisions about discharge of elderly hospital patients mainly about freeing blocked beds? A qualitative observational study. BMJ Open 2:e002027. 10.1136/bmjopen-2012-002027 23166138 10.1136/bmjopen-2012-002027 PMC3533092

[39] Hoffmann T, Jansen J, Glasziou P (2018) The importance and challenges of shared decision making in older people with multimorbidity. PLoS Med 15:e1002530. 10.1371/journal.pmed.1002530 29534067 10.1371/journal.pmed.1002530 PMC5849298

[40] Wong SP, Sharda N, Zietlow KE, Heflin MT (2020) Planning for a safe discharge: more than a capacity evaluation. J Am Geriatr Soc 68:859–866. 10.1111/jgs.16315 31905244 10.1111/jgs.16315

[41] Naughton C, Galvin R, McCullagh R, Horgan F (2023) Comprehensive geriatric assessment-where are we now, where do we need to be in the context of global ageing? Age Ageing 52:afad210. 10.1093/ageing/afad210 37967124 10.1093/ageing/afad210

[42] Uihlein A, Beissel L, Ajlani AH, Orzechowski M, Leinert C, Kocar TD et al (2024) Expectations and requirements of surgical staff for an AI-supported clinical decision support system for older patients: qualitative study. JMIR Aging 7:e57899. 10.2196/57899 39696815 10.2196/57899 PMC11683657

[43] Bradley G, Baker K, Bailey C (2022) Exploring how occupational therapists and physiotherapists evaluate rehabilitation potential of older people in acute care. Br J Occup Ther 85:199–207. 10.1177/03080226211011386 40337082 10.1177/03080226211011386 PMC12033713

[44] Parchmann N, Orzechowski M, Brefka S, Steger F (2025) Evaluation of an AI-based clinical decision support system for perioperative care of older patients: ethical analysis of focus groups with older adults. JMIR Aging 8:e71568. 10.2196/71568 41105877 10.2196/71568 PMC12533935

[45] Kocar TD, Brefka S, Leinert C, Rieger UL, Kestler H, Dallmeier D et al (2025) Deep learning predicts postoperative mobility, activities of daily living, and discharge destination in older adults from sensor data. Sensors 25:5021. 10.3390/s25165021 40871885 10.3390/s25165021 PMC12389988

[46] Parchmann N, Hansen D, Orzechowski M, Steger F (2024) An ethical assessment of professional opinions on concerns, chances, and limitations of the implementation of an artificial intelligence-based technology into the geriatric patient treatment and continuity of care. Geroscience 46:6269–6282. 10.1007/s11357-024-01229-6 38834930 10.1007/s11357-024-01229-6 PMC11493912

[47] Skuban-Eiseler T, Orzechowski M, Denkinger M, Kocar TD, Leinert C, Steger F (2023) Artificial intelligence-based clinical decision support systems in geriatrics: an ethical analysis. J Am Med Dir Assoc 24:1271-1276.e4. 10.1016/j.jamda.2023.06.008 37453451 10.1016/j.jamda.2023.06.008

[48] Quentin W, Geissler A, Scheller-Kreinsen D, Busse R (2010) DRG-type hospital payment in Germany: the G-DRG system. Euro Observer 12:4–7

